# Applications of New Technologies and New Methods in ZHENG Differentiation

**DOI:** 10.1155/2012/298014

**Published:** 2012-05-22

**Authors:** Jianye Dai, Shujun Sun, Huijuan Cao, Ningning Zheng, Wenyu Wang, Xiaojun Gou, Shibing Su, Yongyu Zhang

**Affiliations:** ^1^Research Center for Traditional Chinese Medicine and Systems Biology, Shanghai University of Traditional Chinese Medicine, 1200 Cailun Road, Pudong, Shanghai 201203, China; ^2^Key Laboratory of Liver and Kidney Diseases of Ministry of Education, Shuguang Hospital, Institute of Liver Diseases, Shanghai University of Traditional Chinese Medicine, 528 Zhanghen Road, Pudong, Shanghai 201203, China; ^3^Research Center for Traditional Chinese Medicine Complexity System, Shanghai University of Traditional Chinese Medicine, 1200 Cailun Road, Pudong, Shanghai 201203, China

## Abstract

With the hope to provide an effective approach for personalized diagnosis and treatment clinically, Traditional Chinese Medicine (TCM) is being paid increasing attention as a complementary and alternative medicine. It performs treatment based on ZHENG (TCM syndrome) differentiation, which could be identified as clinical special phenotypes by symptoms and signs of patients. However, it caused skepticism and criticism because ZHENG classification only depends on observation, knowledge, and clinical experience of TCM practitioners, which is lack of objectivity and repeatability. Scientists have done fruitful researches for its objectivity and standardization. Compared with traditional four diagnostic methods (looking, listening and smelling, asking, and touching), in this paper, the applications of new technologies and new methods on the ZHENG differentiation were systemically reviewed, including acquisition, analysis, and integration of clinical data or information. Furthermore, the characteristics and application range of these technologies and methods were summarized. It will provide reference for further researches.

## 1. Introduction

Personalized medicine is looming all over the world, especially following the accomplishment of the Human Genome Project (HGP). Major investments in basic science have created an opportunity for significant progress in clinical medicine. Scientists are developing and using diagnostic tests based on genetics or other molecular mechanisms to better predict patients' responses to targeted therapies [1]. Yet, myriad obstacles must be overcome to achieve these goals.

As a holistic approach attempting to bring the body, mind, and spirit into harmony, TCM may bring personalized medicine to the light in an efficient way. As the essential part of its theory, ZHENG, also called Traditional Chinese Medicine (TCM) syndrome or pattern, is a characteristic profile of all clinical manifestations identified by TCM practitioners and consists of not only the body condition, but also mind and spirit. With the general knowledge of ZHENG and its evolution, TCM emphasizes on early diagnosis and prognosis of diseases, especially preventing its recurrence. In TCM clinical practice, “Treatment based on ZHENG Differentiation” (Bian Zheng Lun Zhi) often gets better effect. For example, He et al. [2] reveal that the effective rate of a combination therapy of two Chinese patent medicines (Glucosidorum Tripterygll Totorum Tablet and Yishenjuanbi Tablet) on rheumatoid arthritis was 53.3%, without ZHENG differentiation. Nevertheless, the effective rate was up to more than 80%, with ZHENG differentiation. Besides, Lu et al. [3] found that the effective rate of biomedical combination therapy (diclofenac, methotrexate, and sulfasalazine) in cold syndrome was much higher than in heat syndrome (*P* < 0.01). After 12-week treatment, the effective rates in patients with cold syndrome and heat syndrome were 51.67% and 29.09%, respectively, but for 24-week treatment, it changed to 88.52% and 57.40%. These researches may suggest that treatment based on ZHENG differentiation could improve the specificity and efficiency in both TCM and Western Medicine. 

Although TCM has been practiced effectively more than 3000 years, ZHENG differentiation is still argued, because it depended on clinical observation and TCM practitioner's experience, which would be subjective and unrepeatable. Since the success of personalized medicine relies on having accurate diagnostic tests that identify patients who can benefit from targeted therapies [1], a great breakthrough in TCM diagnosis with objectivity and repeatability is needed. For this goal, TCM researchers have currently done fruitful works with beneficial technologies and methods, such as literature mining and system biological analysis. Here, the new technologies and the new methods of applied in ZHENG differentiation were reviewed, at the aspects of acquisition, analysis, and integration of clinical data or information, respectively.

## 2. Data Acquisition

As the saying goes, “one cannot make bricks without straw,” a qualitative or a quantitative data is required before ZHENG differentiation. The acquisition of the applicable data using the appropriate technologies or methods is first step.

### 2.1. Qualitative Data Acquisition

The qualitative data is usually got from literatures, epidemiological questionnaire, and parameters by the traditional four diagnostic methods (looking, listening and smelling, asking, and touching). It could be used to describe the characteristics, distribution, and evolution of ZHENG, further to classification.

#### 2.1.1. Literature Retrieval

Like as that we could have a further view standing on the shoulders of predecessors, literature retrieval is undoubtedly a feasible method for researching ZHENG differentiation. A research [4] was performed to probe into the characteristics of ZHENGs and their elements distributions in polycystic ovary syndrome. Literatures from 1994 to 2009 on ZHENGs were retrieved with keyword search and classified, then especially a database was set up by Excel for further analysis based on the collected data. With the help of those, the frequencies of 36 syndromes and their elements have been analyzed. 

It is worthy to say that quality control should be taken seriously in the process of literature collection. And we cannot simply copy the Western standards, for example, Cochrane Statement [5]. An evaluation system for ZHENG differentiation should be established, which is suitable for TCM; otherwise, it will restrict even hold back its development.

#### 2.1.2. Epidemiological Design

Clinical epidemiological study is widely used to acquire data, with the methods of retrospective, cross-sectional, and longitudinal study. With retrospective analysis, diagnostic information of 438 patients with chronic severe hepatitis B (CSHB) was investigated by Peng et al. [6]. The principle signs of TCM syndromes were analyzed by frequency and variable cluster analysis for ZHENG differentiation on three clinical stages. Especially, the research on evolution of “dampness-heat,” “spleen deficiency,” and “blood stasis” may provide assistance for dynamical ZHENG differentiation.

#### 2.1.3. Improvement of Four Diagnostic Methods

As the most important traditional methods, four diagnostic methods (looking, listening and smelling, asking, and touching) have to be developed. As depended on TCM practitioners' observation and clinical experience, the shortcoming of these methods is absence of objective criteria and repeatability. Yue and Liu [7] and Pang et al. [8] have digitalized tongue images using computer technology, to bring tongue observation to semiquantitative measure. And TCM pulse detector was utilized to improve the accuracy and repeatability of pulse diagnosis and provide the data but just feeling [9].

### 2.2. Quantitative Data Acquisition

As above data is all acquired by observation and clinical experience, it is not only qualitative and unrepeatable, but also hard to conduct statistical analysis, pattern recognition, and integration with absence of totally digitalization. Therefore, the acquisition of quantitative data is calling for further progress.

#### 2.2.1. “Omics” Technologies

“Omics” consists of genomics, transcriptomics, proteomics, and metabonomics, with the rapid growth of large-scale detection technologies [10]. It directly focuses on biochemistry networks, pathways, metabolites, and molecule targets of whole bodies, at the top-to-down views. With the features of nondestructiveness, integrity, multitarget, high-throughput, and digitalization, “Omics” technologies may provide feasibility to investigate ZHENG, which would be characterized by multifactor, multiphenotype, and dynamic state. 

Genomics/transcriptomics, also known as global gene expression profiling, is a tool for evaluating gene expression levels of thousands of genes in parallel. Technologies such as gene chip, gene sequencing, and differential display are usually applied. Wu et al. [11] performed genomics to assess the correlation between genetic variations of metabolic genes including PPARD, PPARG, and APM1 and the constitutions. The result suggested that SNP and haplotypes of PPARD, PPARG, and APM1 may underlie the genetic basis of the ZHENG classification. Moreover, gene chip technology was used by Lu et al. [12] to reveal gene expression profiles in CD4^+^ T cells to classify cold and heat syndromes.

Proteomics can be defined as the science and technologies associated with mapping, visualizing, and/or quantitating the expression of all or a majority of the proteins in living systems [13]. Technologies used in proteomics have been around two-dimensional polyacrylamide gels combined with mass spectrometer (MS) or liquid chromatography (LC). With the method of two-dimensional electrophoresis (2DE) combined with matrix-assisted laser desorption/ionization time-of-flight mass spectrometer (MALDI-TOF-MS), Liu et al. [14] evaluated the levels of plasma proteins in health donors and patients with the different ZHENGs of chronic hepatitis B. Objective data was provided for ZHENG differentiation and further to suggest the diagnostic standards and guide the clinical treatment. Wu et al. [15] analyzed the plasma from healthy subjects and patients of coronary heart disease. The result found 3 decreased proteins and 6 increased proteins in blood stasis syndrome, compared with normal group. It suggested that fibrinogen and granzyme might be potential diagnostic biomarkers of blood stasis syndrome in coronary heart disease.

Metabonomics is the study of global metabolite profiles in a biological system (isolated cells, tissue, urine, saliva, blood plasma, etc.) under a given set of conditions [16]. Gas chromatography-mass spectrometer (GC-MS), liquid chromatography-mass spectrometer (LC-MS), and nuclear magnetic resonance (NMR) are widely applied in this area. With the technology of GC/MS, Van Wietmarschen et al. [17] have analyzed the plasma metabolism profiles in patients with cold and heat syndromes of rheumatoid arthritis. They classified the two ZHENGs and got seven differential metabolites. Moreover, using UPLC-QTOF-MS, Sun et al. [18] have analyzed urine samples from liver-Qi invasion patients with premenstrual syndrome. The potential biomarkers and metabolic pathways were found from the metabolic profiles. Furthermore, Liu et al. [19] have detected plasma samples using NMR to explore the dynamic evolution and phase characteristics of phlegm and blood stasis syndromes from the biological features of lipid metabolism.

#### 2.2.2. Physiology and Pathology Detection Technology

Signs, symptoms, and biochemical parameters of patients were collected by Yuan et al. [20] from self-designed questionnaires regarding the four diagnostic methods of TCM. The result suggested that different syndromes have different pathological features. Taking an example, dampness-heat syndrome was characterized by obvious hepatic inflammation, poor synthesis function, and more ascites.

#### 2.2.3. Molecular Biology Detection Technology

The correlation between biochemical indicator and ZHENGs was evaluated by Zhao [21]. Seventy female RA patients with cold or heat syndrome were enrolled in this trial. However, as for the expression of cytokine (TNF-*α*, IL-10, IL-8), clinical inflammatory indexes (ESR), and immune indexes (IgA, IgG, IgM, RF, C3), subjects with heat and cold syndrome showed no significant difference, except CRP.

## 3. Data Mining

For the complexity of biomedicine, it is circumscribed for researches only based on experimental data. Therefore, objective and accurate description of phenomenon and regularity in TCM is getting out from statistical analysis and data mining, drawing assistance from computer technologies. As a multidiscipline fused artificial intelligence, statistics, pattern recognition, and so on, data mining in database is equal to knowledge discovery [22, 23], which is initially utilized for genome designator in biomedicine [24].

### 3.1. Association Rule Mining

Association rule mining is one of the major approaches of data mining and perhaps the most common method of knowledge discovery in unsupervised learning systems [25]. It is used to describe significant associations or correlation relationships among a large set of data items. Especially, Wu et al. [26] associated the gene function from the MEDLNE with TCM literatures. And then they established the relationship between diseases and ZHENGs, combined with validating the relationship between ZHENGs and genes.

### 3.2. Rough Sets Theory

As a new math tool to deal with ambiguous and uncertain information, rough sets theory introduced by Pawlak [27] is applied to get some decisions and classification. By deleting unrelated or unimportant information, it is able to simplify information on the premise of keeping classification ability unchanged. The information of symptoms and signs from 287 posthepatitic cirrhosis patients were collected by Zhang et al. to explore the application of rough sets theory in TCM ZHENG diagnosis. The result showed that this model was meaningful for the diagnosis, with 83% coincidence to main six ZHENGs in TCM [28].

### 3.3. Cluster Analysis

Cluster analysis, an exploring way of classification, could describe a set of multivariate methods and techniques. It is often used to classify data into groups, types, profiles, and so on [29]. With multicenter and large-sample survey, two-step cluster analysis was utilized to study the ZHENG distribution rule of essential hypertension by Gu et al. [30]. Compared with the current ZHENG differentiation criteria, this method could add two more ZHENGs which may be used to reflected etiological factor.

### 3.4. Bayesian Networks

Bayesian network is a kind of probability network which is based on probabilistic reasoning, with the foundation of Bayes formula. Especially through their ability to coordinate bidirectional probabilistic inferences, Bayesian networks are now considered to be a general representation scheme for uncertain knowledge [31, 32]. Qu et al. [33] used Bayesian network to classify ZHENGs in 611 depression patients. The ZHENGs of depression were differentiated by various principle or peripheral ZHENGs and their combinations. The ZHENGs described in their study were in line with clinical TCM and might provide a good guidance for treatment.

### 3.5. Decision Trees

Decision trees are characterized by a logic function which is constant over some box-shaped regions of the X range. These regions are usually represented by a binary decision tree consisting of nodes and binary splits [34]. It can be applied in the development of ZHENG classification. Zhong et al. [35] developed a method of decision trees combined with association rules to study Qi stagnation syndrome in gastritis, getting satisfactory prediction.

### 3.6. Artificial Neural Network

With ability to fitting function at any precision, artificial neural network is powerful to use a structure similar with cerebrum neural synapse to deal with information. It has been demonstrated successfully in many classification tasks [36]. Neural network model trained by conjugate gradient algorithm was built by Sun et al. [37] to classify ZHENGs of coronary heart disease, with 89.2% accuracy. The research got satisfactory results and overcame the shortcomings of traditional BP algorithm effectively.

### 3.7. Principal Component Analysis

Beginning with the interrelation of the variables, principal component analysis based on the dimension reduction is a statistical method that could translate many variables to fewer unrelated integrated variances [38]. Metabonomics based on UPLC/MS had been performed by Lu et al. [39] to study Kidney-Yang deficiency syndrome and therapeutic effect of *Rhizoma Drynariae*. With PCA, a clear separation of model group and predose group was achieved. The time-dependent regression tendency in *Rhizoma Drynariae* treatment group from 1 to 15 days was obtained, which provided a visual, overall, and dynamic progress.

### 3.8. Partial Least Squares Method

Partial least squares (PLSs) method was proposed by Wold, which extracts characteristics based on the principle of maximizing covariance of independent and dependent variable [40]. It makes the characteristics to have much associativity with the dependent variable, improving the precision of the ZHENG classification followed. As clinical samples have more individual variations than animal samples, the supervised methods like PLS are better at concerning the main intergroup difference of clinical samples than unsupervised methods like PCA. Van Wietmarschen et al. [17] used partial least squares-differentiation analysis (PLS-DA) to distinguish cold and heat syndromes of RA patients which were not distinguished by PCA, getting satisfactory result of 3-oxo-propionic acid and other differential metabolites.

### 3.9. Factor Analysis

Factor analysis is used to find the least number of factors to account for the common variance of a large set of statistical expert system variables, excluding variable-specific (unique) variance [41]. It could be applied in analyzing the correlativity of many primitive markers, and then finding out the limited and unobserved potential variance which dominates and explains the correlativity. Multicenter prospective research on TCM ZHENG in 815 cases of unstable angina was conducted by using factors analysis with the nonlinear dimension reduction. Wang et al. [42] suggested that this method could help to classify ZHENG and establish the preliminary diagnostic criteria.

### 3.10. Structural Equation Modeling

Structural equation modeling is based on statistical methodology to study and deal with complex and multivariable data. This technique allowed for the computation of individual measurement errors associated with the observed variables [43]. What is more, it allows testing of a priori hypotheses about the complex causality between the latent variables of diseases and ZHENG**s**. Here, the ZHENGs and domain changes of menopause syndrome on samples of 236 women from literature retrieval were identified by exploratory factor analysis. After finding principle ZHENG of Kidney-Yang and Kidney-Yin deficiency by latent tree, structural equation modeling was applied to confirm the former result [44].

In addition, set pair analysis [45], logistic regression [46], entropy cluster algorithm [47], and support vector machines [48] were applied in ZHENG differentiation with satisfactory results.

## 4. Integration of Data or Bioinformation

An example is shown about how to integrate information. Systems biology approach with the combination of computational analysis and animal experiment was used to investigate this complex issue, ZHENG, in the context of the neuroendocrine immune (NEI) system. By using the methods of literature mining, network analysis, and topological comparison, it was revealed that hormones and immune factors were predominant in the cold and heat syndromes networks, respectively, which were connected by neurotransmitters. In addition, genes related to heat-related diseases are mainly present in the cytokine-cytokine receptor interaction pathway; whereas genes related to cold-related diseases are linked to the neuroactive ligand-receptor interaction pathway. Also, it was in a position to interpret the scientific basis of both ZHENG and associated herbal treatments [49].

The “interaction-network-function” strategy of integration reflecting from “Entity Ontology” to “Relation Ontology” was according to the holism of TCM in methodology.

## 5. Summary and Prospect

With the features of high throughput and multilevel, “Omics,” and bioinformatics technologies are appropriate tools to investigate the holistic characteristics of ZHENG differentiation. In order to easily understand technologies and methods, application range, advantages and disadvantages of “Omics,” and bioinformatics, it was resumptively summarized in Table 1.

To find the characteristics and pathogenesis of ZHENGs through high throughput and multilevel, qualitative, and qualitative data, the data mining methods were applied. The advantage and the disadvantage of these methods were resumptively summarized in Table 2.

Given the limitation of single method and single subject, the multidisciplinary such as mathematics, physics, biology, and statistics would be combined underlying the direction of system theory, which may bring ZHENG researches to an objective and quantized way. For example, cold and heat syndrome has been studied with multiple technologies and methods such as “Omics,” bioinformatics and laboratory index [50]. And Bayesian network, rough set, and generalize connected coefficient were combined to classify ZHENG in liver cirrhosis [28]. And we advocate that systematically combined the appropriate technologies or methods to establish a characteristic “net-marker” of ZHENG differentiation using clinical signs, syndromes, biochemical indicators, and “Omics” data.

**Figure 1:**
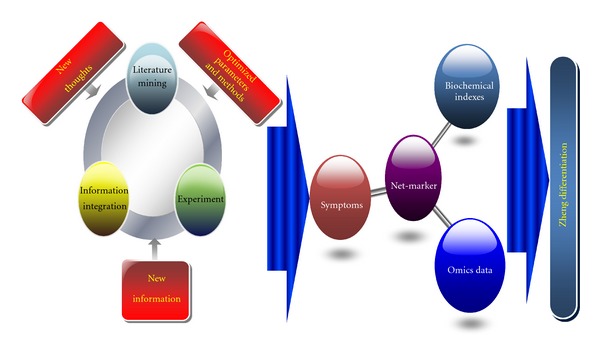
Schematic diagram of research approach for ZHENG differentiation.

Furthermore, we proposed a ZHENG differentiation research approach bases on a computer-aided “information-experiment-information” model (Figure 1). By literature mining, researchers firstly could get necessary information to provide ideas, which include clinical syndromes and signs, laboratorial samples using the suitable methods. The ideas could guide new information which comes from experiments and supply validation. Then, analysis and integration of new data will produce further information for ZHENG differentiation. 

In this progress, assistances are drawn from computer technologies. Data mining could provide the comprehensive and efficient way to deal with the massive data. Suitable methods with broader vision and optimized parameters could be explored by the objective data, but experience and subjective decision. And then, feedback will be got timely from the experiments by powerful statistical analysis, to guide next ones. Furthermore, the “net-marker” acquired from integration of former results may provide an overall and novel understanding of ZHENG for differentiation. The approach shows many differences to traditional thoughts on feasibility and directivity, reducing blindness and consumption (Figure 1).

In addition, for the clinical transformation in ZHENG differentiation, a further research of dynamic changes of ZHENG is needed. Following the development of high-throughout and noninvasive methods, especially the system biological technologies, may give the support to the dynamically differentiating ZHENG. Furthermore, the TCM information and bioinformation would be combined to make TCM syndrome network with the dynamic characteristic by bioinformatics and computer technologies. 

Following the development of new technologies and new methods, the upgrade of TCM researchers' ability, and the expansion of views on the research of ZHENG differentiation, we all believe that objective and accurate approach would be beneficial to TCM diagnosis and treatment. As a result, TCM may play a more important role in personalized medicine.

## Figures and Tables

**Table 1:** Brief introduction of “Omics” and bioinformatics.

Omics	Objects	Technologies and methods	Advantages	Disadvantages	Literatures
Genomics (transcriptomics)	DNA, mRNA	Gene sequence, differential display, subtractive hybridization, EST, SAGE, chip technology	Gene polymorphism Susceptibility for prognosis and treatment Completed database High throughput	Nonassociation to regulation of life activities Nonconsistent strictly with mRNA expression	Wu et al. [11] Lu et al. [12]

Proteomics	Amino acids, protein	Cleaving isotope-coded affinity tag, 2D-MS, 2D-HCLP	Performer of life function	Instability Variability	Liu et al. [14] Wu et al. [15]

Metabonomics	Metabolites	NMR, GC-MS, LC-MS	Amplified action Simplicity to detect Less numbers Similarities in different species	Lack of beneficial supports Interferences by physiological factors	Van Wietmarschen et al. [17] Sun et al. [18] Liu et al. [19]

Bioinformatics	Data, bioinformation	Data mining, network analysis, topological comparison, and so on	Totally holism Exploration of the potential of information Focusing on function relation	Needing of self-development	Li [49]

**Table 2:** Brief introduction of data mining methods.

Methods	Advantages	Disadvantages	Literatures
Logistic regression	Multifunction	Needing of sample size	Luo et al. [46]

Bayesian networks	Utilization of incomplete and inaccurate data	Needing of preceding researches as guidance	Qu et al. [33]

Rough sets theory	Without priori information; simplicity; handling ambiguous and uncertain information	Needing of self-development	Zhang et al. [28]

Association rules mining	Supporting indirect data mining	Nonselectivity; subjectivity	Wu et al. [26]

Set pair analysis	Suitability for changing systems	Handicap in handle relatively precise problems	Li et al. [45]

Structural equation modeling	Analyzing the causality between the latent variables	Needs of 200 samples at least	Chen et al. [44]

Cluster analysis	Minimization errors caused by subjective judgment	Too much calculation; handicap in clustering data with multidimensions and multilevel	Gu et al. [30]

Decision trees	Handling in nonnumeric data; Simplicity	Maybe misleading	Zhong et al. [35]

Principal component analysis	Dimension reduction; holism	Less specificity	Lu et al. [39]

Partial least squares method	Specificity	Handicap in deciding principal component	Van Wietmarschen et al. [17]

Artificial neural network	Simplicity; nonlinear	Handicap in obtaining the hidden information	Sun et al. [37]

Entropy cluster algorithm	Little demand on variances' types; analysis on any statistical dependence of the variances	Needing of self-development	Wang et al. [47]

Factor analysis	Correction capability; views to latent variables	Absence of domination and relationship between primary and secondary	Wang et al. [42]

Support vector machine	Classification without representing the feature space explicitly	Expressing the more complex prior information; analyzing limited samples	Yang et al. [48]
